# White matter, cognition and psychotic-like experiences in UK Biobank

**DOI:** 10.1017/S0033291721004244

**Published:** 2023-04

**Authors:** M. J. Bosma, S. R. Cox, T. Ziermans, C. R. Buchanan, X. Shen, E. M. Tucker-Drob, M. J. Adams, H. C. Whalley, S. M. Lawrie

**Affiliations:** 1 Department of Psychology, University of Amsterdam, Amsterdam, Netherlands; 2 School of Philosophy, Psychology and Language Sciences, University of Edinburgh, Edinburgh, UK; 3 Division of Psychiatry, University of Edinburgh, Royal Edinburgh Hospital, Edinburgh, Scotland, UK; 4 Department of Psychology, University of Texas at Austin, Austin, USA

**Keywords:** Psychosis, psychotic-like experiences, cognition, processing speed, white matter, psychosis and distress

## Abstract

**Background:**

Psychotic-like experiences (PLEs) are risk factors for the development of psychiatric conditions like schizophrenia, particularly if associated with distress. As PLEs have been related to alterations in both white matter and cognition, we investigated whether cognition (g-factor and processing speed) mediates the relationship between white matter and PLEs.

**Methods:**

We investigated two independent samples (6170 and 19 891) from the UK Biobank, through path analysis. For both samples, measures of whole-brain fractional anisotropy (gFA) and mean diffusivity (gMD), as indications of white matter microstructure, were derived from probabilistic tractography. For the smaller sample, variables whole-brain white matter network efficiency and microstructure were also derived from structural connectome data.

**Results:**

The mediation of cognition on the relationships between white matter properties and PLEs was non-significant. However, lower gFA was associated with having PLEs in combination with distress in the full available sample (standardized *β* = −0.053, *p* = 0.011). Additionally, lower gFA/higher gMD was associated with lower g-factor (standardized *β* = 0.049, *p* < 0.001; standardized *β* = −0.027, *p* = 0.003), and partially mediated by processing speed with a proportion mediated of 7% (*p* = < 0.001) for gFA and 11% (*p* < 0.001) for gMD.

**Conclusions:**

We show that lower global white matter microstructure is associated with having PLEs in combination with distress, which suggests a direction of future research that could help clarify how and why individuals progress from subclinical to clinical psychotic symptoms. Furthermore, we replicated that processing speed mediates the relationship between white matter microstructure and g-factor.

## Introduction

Psychotic experiences are not only symptoms of clinically severe psychiatric disorders like schizophrenia, but also occur in the general population with an estimated prevalence of 5–10%, in which case they can be called psychotic-like experiences (PLEs). Although there is variability in the definition of PLEs (Seiler, Nguyen, Yung, & O'Donoghue, 2020), they generally refer to subclinical hallucinations or delusions. Unlike clinical psychotic symptoms, PLEs are usually transient and often not associated with distress. However, people who have PLEs are at higher risk for schizophrenia and other mental health disorders, especially if they are accompanied by distress (Hanssen, Bak, Bijl, Vollebergh, & van Os, 2005). Elucidating the pathophysiology of PLEs with and without distress could therefore help to understand the development of psychotic symptoms and disorders, and inform measures to prevent the transition from subclinical to clinical symptoms.

White matter disturbances in schizophrenia have been consistently reported, with studies finding reduced white matter integrity and connectivity in the whole brain (Alloza et al., 2017; Ashtari et al., 2007; Kelly et al., 2018; Skudlarski et al., 2010, 2013) and in specific tracts (Burns et al., 2003; McIntosh et al., 2008). Studies have also shown white matter integrity deficits in at-risk populations (Cooper, Alm, Olson, & Ellman, 2018; Muñoz Maniega et al., 2008; Roalf et al., 2019; Tang et al., 2019), which include people that do not yet have schizophrenia but experience attenuated symptoms (Cornblatt et al., 2003; Klosterkötter & Schultze-Lutter, 2000). Other research has also found relationships between having PLEs and widespread functional dysconnectivity, altered white matter microstructure or decreased FA in the auditory hemispheric pathway (Oestreich, Randeniya, & Garrido, 2019; O'Hanlon et al., 2015; Orr, Turner, & Mittal, 2014). Furthermore, Schoorl et al. (2021) found a significant association between localized white matter integrity in the splenium of the corpus callosum and a specific PLE (visual hallucinatory experiences), especially when accompanied by distress. Connectome approaches to the study of cognition and PLEs have not yet delivered any consistent results.

Importantly, reduced white matter quality has also been linked to diminished cognitive ability in the healthy population, people with first-episode psychosis and in people with chronic schizophrenia (Alloza et al., 2017; Holleran et al., 2020; Penke et al., 2012a, b; Pérez-Iglesias et al., 2010; Power et al., 2019; Voineskos et al., 2013). Impaired cognitive functioning in domains like memory and executive functions is a core problem in schizophrenia, but also occurs in at-risk populations and in healthy people who report PLEs (Fusar-Poli et al., 2012; Mohamed, Paulsen, O'Leary, Arndt, & Andreasen, 1999; Whyte et al., 2006). Furthermore, longitudinal studies have shown that cognitive deficits are present in people who will later report PLEs or develop schizophrenia (Barnett et al., 2012; Khandaker, Barnett, White, & Jones, 2011; MacCabe, 2008).

The quality of white matter can be assessed through its microstructure, which can be estimated with diffusion MRI (dMRI). In white matter, the directional coherence of water molecular diffusion (known as fractional anisotropy, or FA) is affected by multiple microstructural properties, including axonal myelination, fiber density and axonal ordering (Jones, Knösche, & Turner, 2013). An often used complementary estimate of microstructure is mean diffusivity (MD), an inverse measure of membrane density (Alexander et al., 2011). In addition, the connectome approach provides a more detailed pattern of brain connectivity beyond the major white matter tracts. A whole-brain structural connectome can be constructed *in vivo* from dMRI, resulting in a network formed from nodes (brain areas) and edges (white matter tracts) (Sotiropoulos & Zalesky, 2019). Aspects of network topology can then be computed using graph theory, a technique which characterizes a variety of global properties from these complex networks (Bullmore & Sporns, 2009). It has been proposed that the brain functions effectively because these nodes and edges have the properties of a small-world network, which means that the brain is highly integrated but also maintains some level of segregation (Gong et al., 2009; Griffa, Baumann, Thiran, & Hagmann, 2013). This high level of integration is thought to be reduced in schizophrenia, often called a ‘dysconnectivity’ disorder. In accordance, several studies have found that a graph-theory measure of network efficiency, called global efficiency, was reduced in participants with schizophrenia compared to controls (Griffa et al., 2013).

Reduced network efficiency is also associated with less effective cognitive functioning – in terms of both general cognitive ability (Li et al., 2009; Van Den Heuvel, Stam, Kahn, & Hulshoff Pol, 2009) and processing speed (Pineda-Pardo, Martínez, Román, & Colom, 2016; Wen et al., 2011). Moreover, processing speed is a key mechanism for higher-order cognition and therefore general cognitive ability (Kail & Salthouse, 1994). Indeed, studies have shown that the relationship between white matter integrity and general intelligence is (partially) mediated by processing speed in healthy participants (Penke et al., 2012a, b) and in people with schizophrenia (Alloza et al., 2016).

**Fig. 1.**
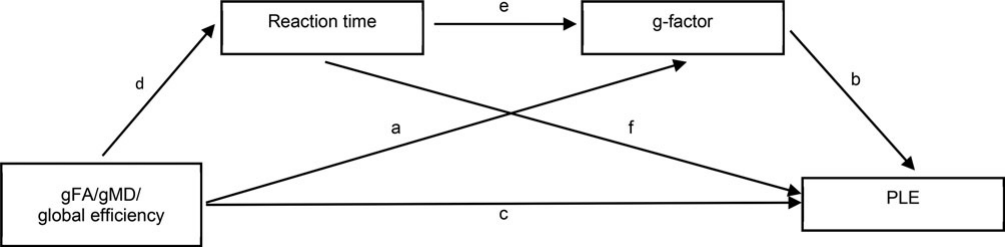
Path diagram of the model that was tested. *Note.* Path diagram specifies that the association between ostensibly poorer (higher MD, lower FA and lower global efficiency) white matter metrics and having PLEs is mediated via processing speed and general cognitive ability. Though prior work suggests that processing speed may underpin g, we also allow for a separate independent path directly from speed to PLEs.

Given the well-established relationships between white matter and both processing speed and general cognitive functioning, the observation of reduced cognitive functioning in people with PLEs and the previously found association between white matter microstructure and PLEs, we sought to investigate a comprehensive framework of the relationships between white matter, cognition and PLEs. We hypothesized that general cognitive ability mediates the relationship between white matter microstructure or network efficiency and reporting PLEs. Further, aiming to replicate the findings of Penke et al. (2012a, b), we hypothesized that processing speed acts as an intermediate mediator between white matter properties (white matter microstructure and network efficiency) and general cognitive ability. These hypotheses were investigated through path analysis, first in a population-based sample of *N* = 6170 participants who had detailed (connectomic) data available. We then tried to replicate these findings with less detailed white matter indicates in a larger population-based sample of *N* = 19 891 participants. The model that was tested can be seen in Fig. 1. The preregistration of this study can be found on https://osf.io/2jkty and deviations from this protocol are reported in the supplementary materials.

## Methods

### Sample

The data were provided by UK Biobank (UKB), a cohort of approximately 500 000 middle-aged and older community-dwelling participants from the United Kingdom and Northern Ireland, under project number 4844 linked to projects 10 279 and 16 124. More information on the recruitment of participants and their sociodemographic and health-related characteristics can be found in Fry et al. (2017) and on http://www.ukbiobank.ac.uk/key-documents/. UKB has received ethical approval from the Research Ethics Committee (reference 11/NW/0382) and all participants provided informed consent.

A head MRI scan was available in a subset of participants. 6170 of these participants completed the online Mental Health Questionnaire and had individual connectomes constructed following the methods of Buchanan et al. (2020). This sample will henceforth be referred to as the connectome sample. Twenty of the participants in this sample reported having a psychotic disorder (F20 – F29 in ICD-10). The results of all analyses reported below are excluding these individuals, but analyses with these individuals included are reported in online Supplementary Tables S11–S16, S19, S20, S23, S24 in the supplementary materials.

**Fig. 2.**
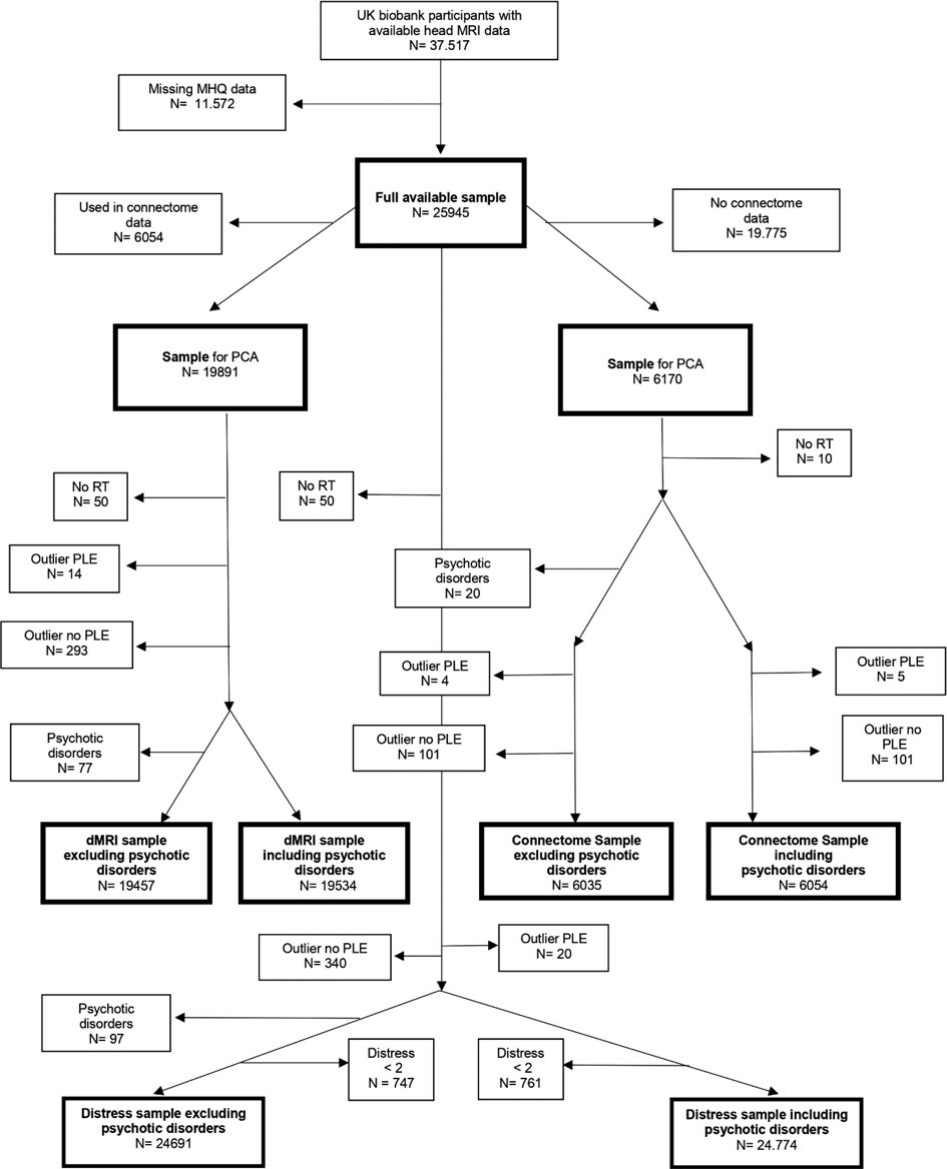
Participant flow chart.

To try to replicate any findings in a larger sample, we also performed all analyses on participants who had UKB processed diffusion MRI (dMRI) data available from a head MRI scan and who completed the Mental Health Questionnaire. This resulted in a sample of *N* = 25 945 participants with dMRI but no connectome data. Since this sample overlapped with the connectome sample, we removed all participants that were also in the connectome sample. This resulted in a sample of *N* = 19 891, which we will henceforth call the dMRI sample. Seventy seven of the participants in this sample reported having a psychotic disorder (F20 – F29 in ICD-10). See Fig. 2 for a flowchart of the participants.

### Materials

Details about the exact data fields and a link to the corresponding data dictionary can be found in the supplementary methods, online Supplementary Table S1.

#### Cognitive tests

To assess general intelligence and processing speed, we used results from the UKB cognitive battery (for a description of the used tasks, see supplementary materials). This battery does not include any standardized tests, but a recent study concluded that their concurrent validity and short-term test-retest reliability was moderate to good (Fawns-Ritchie & Deary, 2020). The results regarding the validity and reliability of the measures that were used can be found in the supplementary methods. We used the score on the UK Biobank reaction task (RT, field ID 20023 http://biobank.ndph.ox.ac.uk/showcase/field.cgi?id=20023) to assess processing speed. To create a measure of general cognitive ability, the test scores on UKB pairs memory (field ID 399, http://biobank.ndph.ox.ac.uk/showcase/field.cgi?id=399), prospective memory (field ID 20018, http://biobank.ndph.ox.ac.uk/showcase/field.cgi?id=20018), fluid intelligence (field ID 20016, http://biobank.ndph.ox.ac.uk/showcase/field.cgi?id=20016) and numeric memory (field ID 4282, http://biobank.ndph.ox.ac.uk/showcase/field.cgi?id=4282) were entered in a principal component analysis (PCA).

#### Psychotic-like experiences

PLEs were assessed with a Mental Health Questionnaire (MHQ; UKBiobank Category: 144. Four different questions asked whether participants had ever had a visual or auditory hallucination, persecutory delusion or delusion of reference. We merged the people who answered yes to at least one of these questions into one group, and thus ended up with two groups: participants with at least one PLE and participants without any PLE. Finally, the participants were asked how distressing they found their experience, to create a variable that was used in the exploratory analyses. Details of this questionnaire can be found in the supplementary methods, online Supplementary Table S2.

#### Imaging protocol and connectome derived variables

T1 weighted and diffusion MRI images were acquired using a Siemens Skyra 3 T scanner. More detailed information on imaging acquisition, preprocessing and quality control can be found in UK Biobank (2014) and in Alfaro-Almagro et al. (2018). Cortical reconstruction and segmentation were conducted in-house using FreeSurfer 5.3 with default settings, and visual quality control procedures to identify and remove major processing fails, skull strip errors and tissue segmentation issues. Each T1 weighted image was segmented into 85 regions of interest (ROI). Cortical ROIs were consequently identified according to the Desikan−Killiany atlas (Desikan et al., 2006), resulting in 34 cortical structures per hemisphere. Eight subcortical gray matter ROIs per hemisphere and the brain stem were also identified based on Fischl et al. (2002).

Subsequently, brain structural connectomes were derived. Whole-brain probabilistic tractography was performed and weighted connectomes were constructed by identifying all connections between ROIs. The network weightings were computed for FA and MD and recorded in 85 × 85 connectivity matrices. Further information on the construction of the connectomes can be found in Buchanan et al. (2020). We applied a consistency thresholding method (Roberts, Perry, Roberts, Mitchell, & Breakspear, 2017) to retain 30% of all connections with weights that were consistent across subjects (Buchanan et al., 2020).

The weights of all individual network connections which survived the consistency threshold were entered in a PCA to obtain general FA (gFA) and general MD (gMD) as measures of the first independent variable; white matter microstructure (Alexander et al., 2011). The graph theory metric global efficiency, the inverse of the average shortest path length between all pairs of nodes in the network (Rubinov & Sporns, 2010), was calculated from both weighted networks. This resulted in FA derived global efficiency (GeFA) and MD-derived global efficiency (GeMD), as measures of the second independent variable, network efficiency.

#### UKB imaging derived phenotypes (IDPs) variables

UK Biobank provides minimally processed, ready-to-use measures of white matter integrity, called imaging derived phenotypes (IDPs). We used the weighted FA and MD values of 27 white matter tracts derived through diffusion MRI probabilistic tractography to obtain a second set of general FA and MD measures, which will henceforth be referred to as the IDP-derived gFA and gMD measures. Further information on how the IDPs (27 tracts) derived gFA and gMD measures where constructed can be found in http://biobank.ctsu.ox.ac.uk/crystal/crystal/docs/brain_mri.pdf.

### Statistical analyses

All statistical analyses were performed in R, version 3.5.3 and version 3.6.1.

#### Principal component analyses

To account for missing values in cognitive measures, individual scores were imputed using the missMDA package (Josse & Husson, 2016). A PCA was then performed on the four cognitive variables and the scores on the first unrotated component (accounting for ~43% of the total variance, loadings all >0.30) were extracted as a measure of general cognitive ability (g). Details on the results of this PCA can be found in the supplementary materials online Supplementary Table S3 and online Supplementary Figs. S2–S3. We also performed a PCAs on the IDP derived variables and extracted the first unrotated component of FA and MD (both accounting for ~40% of total variance) as measures of IDP derived gFA and gMD. Details on the results of these PCAs, can be found in the supplementary materials online Supplementary Figs. S6–S9 and online Supplementary Table S4. The above mentioned PCAs were performed separately in all samples. Finally, we performed PCAs on the FA and MD weighted white matter network connections which remained after network thresholding (Madole et al., 2020). The scores of the first unrotated component for FA and MD were extracted as a measure of gFA and gMD (respectively accounting for 18.3% and 22.4% of the total variance). Details on the results of these PCAs can be found in supplementary materials online Supplementary Figs. S4–S5.

#### White matter analyses

First, we performed a log transformation on the reaction time task scores and excluded participants that did not have any score on this task. We then identified and excluded participants who had scores of 3.5 standard deviations above or below the group mean (having PLE or not) for all derived predicting and mediating variables (reaction time, g-factor, gFA, gMD, GeFA, GeMD, IDP derived gFA and IDP derived gMD). The extreme scores on the imaging variables were removed because we believe that they mostly represented artifacts and processing errors that can be missed by the automated quality control pipeline. We also chose to exclude extreme values on the cognitive variables that might have arisen due to the unsupervised nature of the cognitive tests.

Next, we tested the model proposed in the introduction, using path analysis with lavaan version 0.6–5 (Rosseel, 2012). In this model, we specified that the association between ostensibly poorer white network efficiency/microstructure and having PLEs is mediated via processing speed and general cognitive ability (see Fig. 1). Though previous research suggests that processing speed may underpin g, we also allowed for a separate independent path directly from processing speed to PLEs. Following our hypotheses, we were especially interested in the possible significance of the total effects of white matter measures on PLE (path c + (a×b) + (d×f) + (d×e×b)) and white matter measures on g (path a + (d×e)) and the confidence intervals of the indirect effects through cognition and processing speed (d×e and (a×b) + (d×f) + (d×e×b)). The exact defined model can be found in supplementary methods online in Supplementary Fig. S9. Per analysis, only the predicting variable changed between connectome derived gFA, gMD, GeMD, GeFA and IDP derived gFA and gMD. Age and sex were included as covariates and we used diagonal weighted least squares (DWLS) as an estimator because of the dichotomous nature of the outcome variable (PLE). Due to the asymmetrical nature of the sampling distribution of the indirect effect, given that they involve the multiplication of other effects, we also calculated a thousand iterations bootstrapped confidence intervals for all estimates (Bollen & Stine, 1990). This whole process was repeated in the dMRI sample with only IDP derived gFA and gMD. Here scan site was included as an extra covariate because the dMRI sample was acquired on multiple scanners whereas the smaller connectome sample was acquired on a single scanner.

#### Distress analyses

We also wanted to explore the role of distress in our analyses. Therefore, we performed exploratory analyses to test the model in the full sample available (*N* = 24 774), with only participants who indicated to have felt a little or more distress associated with their PLEs (a score of 2 or higher on the distress question) included in the PLE group. In these analyses, participants with distress and PLEs were coded as one, and participants without PLEs were coded as 0. Before analysis, outliers were removed in the same fashion as in the other samples. Because of low power due to few participants reporting a distress score of 2 or higher, we only performed these analyses in the full available sample.

All *p* values were corrected for false-discovery rate (FDR) (Benjamini & Hochberg, 1995) over the different samples that were tested and all reported betas are standardized. We corrected for different paths and different white matter measures separately, meaning that we corrected different FA measures over all samples, different MD measures over all samples etc. For example, we corrected for the FA measures of the path ‘a’ over all confirmatory analyses, so we performed FDR correction over the six *p* values (FA connectome, FA IDP and FA including and excluding participants with a psychotic disorder).

## Results

### Descriptive statistics

We excluded 20 participants with psychotic disorders and 105 outliers from the connectome sample. From the dMRI sample, we excluded 77 participants with psychotic disorders and 315 outliers. Details on the final sample size, demographics and descriptive statistics of all participants can be found in Table 2.

**Table 1.** Table indicating outcomes of interest per hypothesis

Hypothesis	Outcome of interest	Indicates
H1	c + (a×b) + (d×f) + (d×e×b)	Total effect of white matter on PLE
	(a×b) + (d×f) + (d×e×b)	Indirect effect of WM on PLE, through g and processing speed
H2	a + (d×e)	Total effect of white matter on g-factor
	d×e	Indirect effect of WM on g-factor, through processing speed

**Table 2.** Demographics and descriptive statistics of participants, divided per sample and group.

Variable	Unit	Connectome sample			dMRI sample		
		No PLE	PLE	*p* value	No PLE	PLE	*p* value
Count	Number	5749	286	–	18 564	893	–
Age	Years (s.d.)	55.32 (7.4)	54.29 (7.8)	0.026^a^	54.76 (7.4)	53.85 (7.2)	0.001^a^
Sex	F (% females)	3081 (53.6)	179 (62.4)	0.004^b^	10 136 (54.6)	537 (60.2)	<0.001^b^
G-factor	Score (s.d.)	0.046 (1.18)	−0.048 (1.22)	–	0.055 (1.2)	0.025 (1.3)	–
Log RT	Score (s.d.)	6.274 (0.17)	6.266 (0.17)	–	6.264 (0.16)	6.269 (0.17)	–
GeFA	Unit (s.d.)	0.304 (0.01)	0.304 (0.01)	–	–	–	–
GeMD	Unit (s.d.)	0.0005 (1.8 × 10^−5^)	0.0005 (1.8 × 10^−5^)	–	–	–	–
gFA	Unit (s.d.)	0.015 (0.74)	0.019 (0.74)	–	–	–	–
gMD	Unit (s.d.)	−2.5 × 10^−5^ (0.001)	−1.9 × 10^−5^ (0.001)	–	–	–	–
IDP gFA	Unit (s.d.)	0.0012 (0.07)	0.0006 (0.07)	–	0.0016 (0.076)	−0.0008 (0.08)	–
IDP gMD	Unit (s.d.)	−2.1 × 10^−6^ (0.0001)	−8.2 × 10^−6^ (0.0001)	–	−4 × 10^−7^ (0.0001)	−2.7 × 10^−6^ (0.0001)	–

*Note.* Abbreviations: GeFA = FA derived global efficiency, GeMD = MD derived global efficiency, gFA = general FA, gMD = general MD, log RT = log reaction time, IDP = Imaging derived phenotype.

a Derived through two-sample *t* test;.

b Derived through chi-square test.

### Psychotic-like experiences

Below, we will refer to our outcomes of interest as total and indirect effects; for the exact specifications see Table 1. All analyses below are excluding participants with a psychotic disorder. Results of all analyses, also containing analysis including participants with psychotic disorder, can be found in supplementary materials online Supplementary Tables S5–S20.

Our analyses showed no significant total effects between PLEs and network efficiency or white matter microstructure in all the samples tested (*β* < 0.046, *p* > 0.30). The indirect effects, as an indication of the mediation effect of cognition on the relationship between white matter variables and PLEs were also non-significant (*β* < 0.0012, *p* > 0.35). More details on the results of these analyses can be found in supplementary materials, online Supplementary Tables S5–S20.

Importantly, we also ran the exploratory path analysis in the full available sample with only participants included in the PLEs group that also reported distress score >1 associated with their PLEs. There, we found that those with distressing PLEs had significantly lower gFA (*β* = −0.055, *p* = 0.008). However, they did not have significantly lower cognitive functioning (*β* = −0.0322, *p* = 0.2277) and there was no evidence of any mediation effect (*β* = −0.002, *p* = 0.1203). Tables with output from the above analyses can be found in supplementary materials, online Supplementary Tables S21–S24.

### White matter and cognition

In the connectome sample, analyses showed that higher g-factor was significantly related to lower IDP derived gMD (*β* = −0.03, *p* = 0.040) and higher IDP derived gFA (*β* = 0.042, *p* = 0.026). This same effect was found in the dMRI sample, with IDP derived gMD (*β* = −0.027, *p* = 0.003) and with IDP derived FA (*β* = 0.049, *p* < 0.001). This means that there was a significant total effect of the IDP derived white matter variables on g-factor.

Another effect that replicated across samples was the relationships between lower gMD, higher gFA derived from both connectome or IDP derived data and slower reaction time, with absolute beta's ranging from *β* = 0.0272 to *β* = 0.048 and *p* values ranging from *p* *=* 0.036 to *p* < 0.001. Higher MD derived global efficiency also related significantly to slower reaction times (*β* = 0.049, *p* = 0.001).

Further, analyses showed that faster reaction time was related to higher g-factor in the connectome sample (*β* = −0.10, *p* < 0.001) and the dMRI sample (*β* = −0.08, *p* = < 0.001).

Because the direct effect and both indirect effects of the mediation model proved to be significant when tested with IDP derived gFA and gMD, we evaluated whether the relationship between both white matter parameters and g-factor was indeed partially mediated by reaction time. In support of our hypothesis, the confidence intervals of the indirect effects did not contain zero in both samples, indicating partial mediation. In the connectome sample, the proportion mediated was 8.3% (*p* = 0.020) for gFA and 15% (*p* = 0.005) for gMD. This effect replicated in the dMRI sample, with a proportion mediated of 7% for gFA (*p* < 0.001) and 11% (*p* < 0.001) for gMD. In summary, we found a total effect of higher gFA and lower gMD related to an increase in g-factor. We also found significantly higher gFA and lower gMD related to faster reaction times, faster reaction times related to higher g-factor and a significant indirect effect, all indicating a partial mediation.

## Discussion

### White matter and psychotic-like experiences

The results of the confirmatory analyses showed no support for the relationship between white matter microstructure/network efficiency and PLEs, and thus for our hypotheses about the mediating effect of general cognitive ability on this relationship. One possible explanation could be that unlike clinical psychotic symptoms, PLEs are transient and often of low impact on the person who experiences them. It is thus plausible that white matter disturbances are minimal in people with PLEs, especially in our relatively old UKB sample. Furthermore, the mean age of around 55 in the current sample is well beyond the peak age of developing a psychotic disorder (Hafner et al., 1994), which suggests that the PLEs experienced by people in this sample are probably less likely to have high clinical significance. This reinforces the view that PLEs in this sample may not severe enough to be associated with marked white matter disturbances.

Importantly, we did find that participants in the PLEs group who had experienced distress in relation to their PLEs showed significant relationships between lower gFA and PLEs. This relationship was not mediated by cognition. It does suggest that people who experience PLEs may have altered white matter microstructure compared to people who do not experience PLEs, but especially or perhaps only if these PLEs are severe and/or long enough to cause distress. This association may reflect a neurodevelopmental vulnerability to more severe/uncontrollable PLEs. However, it is also possible that distress in combination with PLEs represents an individual's general tendency toward maladaptive emotion regulation (Osborne, Willroth, DeVylder, Mittal, & Hilimire, 2017) or other psychopathology (Brañas, Barrigón, Lahera, Canal-Rivero, & Ruiz-Veguilla, 2017), instead of the occurrence of more severe PLEs. Regardless, the current finding is in line with previous studies showing that distress associated with PLEs is an important predictor for progression to more severe mental health issues (Hanssen, Bijl, Vollebergh, & Os, 2003). It is also in keeping with previous studies in high-risk populations and participants with psychotic disorders, which have reported associations between white matter microstructure and psychotic symptoms (Alloza et al., 2017; Cooper et al., 2018; Roalf et al., 2019; Skudlarski et al., 2010, 2013; Tang et al., 2019). Notably, it was previously found that the association between white matter integrity in the forceps major and visual hallucinations was stronger in people who reported distress (Schoorl et al., 2021).

Together, these results suggest that distress is an important factor when investigating neurobiological markers of PLEs. Since people who experience distress are more likely to develop a diagnosable psychotic disorder (Calkins et al., 2017), it is possible that their white matter disturbances are more evident than those of people without distress. However, we cannot, at this stage say whether distress may be a marker of content, severity or impact. This should be examined in future studies of younger participants who experience distress associated with their PLEs, or in participants who have a psychotic disorder.

### Cognition

Our analyses showed that the quality of white matter microstructure was associated with a higher general cognitive ability score. This finding is in accordance with a study by Cox, Ritchie, Fawns-Ritchie, Tucker-Drob, and Deary (2019), who also found an association between white matter macro- and microstructure and g-factor, in a UKB sample with an enhanced cognitive battery. Importantly, we also found that higher FA/lower MD was associated with quicker reaction time and that quick reaction time was related to a higher general cognitive ability score. We thus conclude that our results are in keeping with the hypothesis that altered white matter microstructure is associated with lower general cognitive ability, and that this relationship is partially mediated by processing speed. We thus replicate the results by (Penke et al., 2012a, b), who found this same mediation effect in a smaller sample. Our study, therefore, adds to the certainty and universality of the mediation effect of processing speed on the relationship between white matter microstructure and general cognitive ability.

### Limitations

Several limitations should be taken into account when interpreting our results. First of all, we note that all the effect sizes found in our study are small, possibly reducing their clinical relevance. However, small effect sizes are common in large population studies and likely represent reliable, true effects rather than unreliable big effects often found in smaller studies (Button et al., 2013). Further, it is important to mention that FA and MD are not direct measures of white matter quality; rather, they measure multiple microstructural properties, including axonal myelination and fibre density (Jones et al., 2013). The interpretation of reduced or increased gFA and gMD is therefore not straightforward and one should consider this when interpreting a decrease of gFA or an increase of gMD as a sign of white matter disruption (Alexander et al., 2011). Additionally, network variables are known to be sensitive to the network construction methodology used (Qi, Meesters, Nicolay, ter Haar Romeny, & Ossenblok, 2015), which may affect the generalizability of our findings to other samples. Lastly, we chose our graph theory metric (global efficiency) a priori, but realize that other graph theory metrics might have been similarly, or even more predictive of our outcome variables.

We should also note several limitations about the cognitive measures that were used. First of all, due to restricted cognitive data available in initial UKB samples, we only used one test score as an indication of the latent construct processing speed. Ideally, latent constructs should be determined based on at least three indicators. However, research by Fawns-Ritchie and Deary (2020) does show that the UKB reaction time test correlated moderately to highly with other reference tests of processing speed. Secondly, the cognition tasks we used to infer general cognitive ability had relatively low test-retest reliability. Low test-retest reliability means more random error in a variable, causing the variable to relate less strongly to other variables. This limitation is partially mitigated by our latent variable approach. Namely, research by Fawns-Ritchie and Deary (2020) showed that the first principal component of a PCA over five cognitive measures (including the measure for processing speed) correlated highly (*r* = 0.74) with a reference test for the assessment of general intelligence. However, this limitation could still have contributed to some of the null findings regarding the white matter and general cognitive ability. Finally, due to a lot of missing values on the cognition tasks, we have imputed scores before executing the PCA to derive a g-factor, which could have led to an inflated explained variance. However, we made sure to also perform the PCA without imputed values and the explained variance and loadings were still satisfactory (see supplementary results, online Supplementary Table S3 for details).

We have also used data from self-report questionnaires to establish whether participants had PLEs, whether they experienced distress and whether they have been diagnosed with a psychotic disorder. For the PLE and psychotic disorder variables, this yielded binary scores. In future studies, a more precise clinician rating could yield scores with a wider range of values, therefore creating a higher variance in the variable. This would allow for a more reliable indication of these phenomena and a more rigorous and generalizable test (Lakes, 2013) of the relation between white matter and PLEs. Related to this, an interesting suggestion for further research would be to include the amount of PLEs participants experience in the analyses.

The relatively old age of our sample should also be taken into account when interpreting the results of our study. Apart from the fact that our study participants are well beyond the peak age of developing psychosis, the older age could also have influenced the results of the Mental Health Questionnaire. The Mental Health Questionnaire asked questions about experiences that could have occurred over 30 years ago. It is thus plausible that some participants forgot about a PLE that they experienced a long time ago, or about the exact circumstances, such as drug use or distress, surrounding the PLE. However, results from the Mental Health Questionnaire show that approximately 5% of the participants indicated that they had experienced a PLE. Since this is in accordance with a previously reported prevalence of PLE (Van Os, Linscott, Myin-Germeys, Delespaul, & Krabbendam, 2009), it does not appear to have been influenced by the sub-optimal memory of the participants.

Lastly, it is possible that psychotropic medication could have confounded our findings; e.g. that white matter differences could have been linked to psychotropic medication use instead of having PLEs with distress. However, we note that similar findings were reported including and excluding the small number of participants most likely to be taking medication, i.e. those with a diagnosis of a psychotic disorder (97 out of 24 774).

## Conclusion

To conclude, this study presents a novel investigation of the relationships between white matter quality, cognition and PLEs; which has given new insights into the possible importance of distress in these relationships, as well as the validity of connectome measures. Furthermore, we corroborated earlier findings of the mediating role of processing speed on the relationship between white matter microstructure and general cognitive ability, in a big general population sample. Most importantly, we showed that PLEs in combination with distress are associated with reduced global white matter microstructure, which suggests a direction of research that could clarify how and why individuals progress from subclinical to clinical psychotic symptoms.

## Supplementary material

For supplementary material accompanying this paper visit https://doi.org/10.1017/S0033291721004244. click here to view supplementary material
