# Supplementary material for: White matter, cognition and psychotic-like experiences in UK Biobank
Source: Psychol Med. 2021 Nov 17;53(6):2370–9. doi: 10.1017/S0033291721004244 (PMC10123836; doi:10.1017/S0033291721004244)
Supplement: Supplementary file 1 [file S0033291721004244sup001.docx]

**Deviations from preregistration**

We preregistered two research questions, but have chosen to publish these research questions as separate studies. The current study relates to research question 1. All our hypotheses have remained unchanged.

**Sample**

Since a significant amount of participants had not finished the Mental Health Questionnaire, our initial sample consisted of 6170 instead of 8185 participants. In a post-hoc addition to the pre-registered study design, we opted to conduct the prespecified analyses on the larger sample (N = 19775) which had dMRI IDPs (in addition to the smaller connectome-only sample).

**Materials**

We also decided to derive gFA and gMD measures from UKB IDPs as measures to validate and replicate the results on the connectome derived variables.

**Analyses**

Due to a lot of missing values on the numeric memory task, we decided to impute the missing values before performing the PCA on the cognitive task results. Because our predicting variable was dichotomous, we could not use the full information maximum likelihood estimation as intended. Instead, we used the diagonal weighted least squares to estimate our model.

**Supplementary Methods**

**Materials**

*Cognitive tests*  We used the score on the UK reaction task (RT) to asses processing speed. This test correlated moderately with its chosen reference tests, the Deary-Liewald Reaction Time (DLRT) simple (r = 0.52) and DLRT choice (r = 0.43) and it had moderate test-retest reliability (r = 0.550). To create a score for general cognitive ability the test scores on UKB pairs memory, UKB prospective memory, UKB fluid intelligence and UKB numeric memory were entered in a principal component analysis (PCA). The first unrotated component of this PCA was used as a measure of general cognitive ability. In a previous study, the scores on the first unrotated component of a PCA that also included reaction time proved to have a high correlation (r= 0.74) with a reference test for the assessment of general intelligence (Fawns-Ritchie & Deary, 2019).

*UKB Pairs Memory*

During this task, the participants had to memorize the location of 6 pairs of cards that were arranged in 4 rows and 3 columns. This task is similar to the children’s game of memory.

*UKB Prospective Memory*

Participants where asked, early on in the cognitive battery: "At the end of the games we will show you four coloured shapes and ask you to touch the Blue Square. However, to test your memory, we want you to actually touch the Orange Circle instead."

*UKB fluid intelligence*

During this task, participants had to answer as many questions that require logic and reasoning in two minutes.

*UKB Numeric Memory*

For this task, participants had to enter a row of numbers they had just seen on the screen. The task started with a two digit row and became longer by one digit every time the participant answered correctly. Score on this task was the maximum digits remembered correctly by the participant.

*UKB imaging derived phenotypes (IDPs)* The UKB IDPs where previously derived through an automated pipeline that first performed pre-processing and quality control (QC) on the raw MRI data. More information on this pipeline and the background of the UKB imaging study can be found in Alfaro-Almagro et al. (2018) and Miller et al. (2016).

**Table S1**

Data fields and links to the UK biobank data showcase

| **Variable** | **Field ID** | **Data Showcase Link** |
| --- | --- | --- |
| Age (at recruitment) | 21022 | <http://biobank.ndph.ox.ac.uk/showcase/field.cgi?id=21022> |
| Sex | 31 | <http://biobank.ndph.ox.ac.uk/showcase/field.cgi?id=31> |
| UKB reaction task | 20023 | <http://biobank.ndph.ox.ac.uk/showcase/field.cgi?id=20023> |
| UKB pairs memory | 399 | <http://biobank.ndph.ox.ac.uk/showcase/field.cgi?id=399> |
| UKB prospective memory | 20018 | <http://biobank.ndph.ox.ac.uk/showcase/field.cgi?id=20018> |
| UKB fluid intelligence | 20016 | <http://biobank.ndph.ox.ac.uk/showcase/field.cgi?id=20016> |
| UKB numeric memory | 4282 | <http://biobank.ndph.ox.ac.uk/showcase/field.cgi?id=4282> |
| Auditory hallucination | 20463 | <http://biobank.ndph.ox.ac.uk/showcase/field.cgi?id=20463> |
| Number of times (AH) | 20465 | <http://biobank.ndph.ox.ac.uk/showcase/field.cgi?id=20465> |
| Visual hallucination | 20471 | <http://biobank.ndph.ox.ac.uk/showcase/field.cgi?id=20471> |
| Number of times (VH) | 20473 | <http://biobank.ndph.ox.ac.uk/showcase/field.cgi?id=20473> |
| Persecutory delusions | 20468 | <http://biobank.ndph.ox.ac.uk/showcase/field.cgi?id=20468> |
| Number of times (PD) | 20470 | <http://biobank.ndph.ox.ac.uk/showcase/field.cgi?id=20470> |
| Delusion of reference | 20474 | <http://biobank.ndph.ox.ac.uk/showcase/field.cgi?id=20474> |
| Number of times (DoR) | 20476 | <http://biobank.ndph.ox.ac.uk/showcase/field.cgi?id=20476> |
| Distress | 20462 | <http://biobank.ndph.ox.ac.uk/showcase/field.cgi?id=20462> |

**Table S2**

Questions, answer possibilities and coding of the Mental Health Questionnaire

| **Question** | **Answers possibilities** | **Coded as** |
| --- | --- | --- |
| Did you ever believe that there was an unjust plot going on to harm you or to have people follow you, and which your family and friends did not believe existed? | Yes/No | Persecutory Delusions |
| Did you ever believe that a strange force was trying to communicate directly with you by sending special signs or signals that you could understand but that no one else could understand (for example through the radio or television)? | Yes/No | Delusions of Reference |
| Did you ever hear things that other people said did not exist, like strange voices coming from inside your head talking to you or about you, or voices coming out of the air when there was no one around? | Yes/No | Auditory Hallucinations |
| Did you ever see something that wasn't really there that other people could not see? | Yes/No | Visual Hallucinations |
| How distressing did you find having any of these experiences? | “Prefer not to answer”, “Do not know”, “Not distressing at all, it was a positive experience”, “Not distressing, a neutral experience”, “A bit distressing”, “Quite distressing”, and “Very distressing” | 0 (not distressing at all) to 4 (very distressing). |

**Statistical analyses**

*PCA cognition* First, we checked the normality of the four cognitive tests that were used to derive the measure of general cognitive ability. The scores on the pairs memory task were not normally distributed, so we performed a log+1 transformation on this variable. Because of a high number of missing values on the numeric memory and fluid intelligence tasks, we imputed values using the imputePCA function from the missMDA package. The fully imputed dataset without missing values was subsequently scaled and entered in a PCA, where the individual loadings on the first unrotated component was computed. This score was used as a measure of general cognitive ability. This process was repeated for the connectome, dMRI and full available sample.

To check whether the imputation of values did not influence the results of the PCA too much, we also conducted a PCA with only complete cases. This yielded a first principal component that accounted for 0.36% of the total variance. The loadings of the subtests on this principal component can be found in table S3.

**Table S3**

*Loadings of cognitive subtests on first principal component, per sample*

| Subtest | Loadings | |  |  |
| --- | --- | --- | --- | --- |
|  | Connectome sample | dMRI sample | Complete cases |  |
| Fluid Intelligence  Numeric Memory  Prospective Memory  Pairs Matching | 0.539  0.542  0.518  -0.383 | 0.575  0.547  0.527  -0.301 | 0.64  0.61  0.25  -0.39 |  |

**
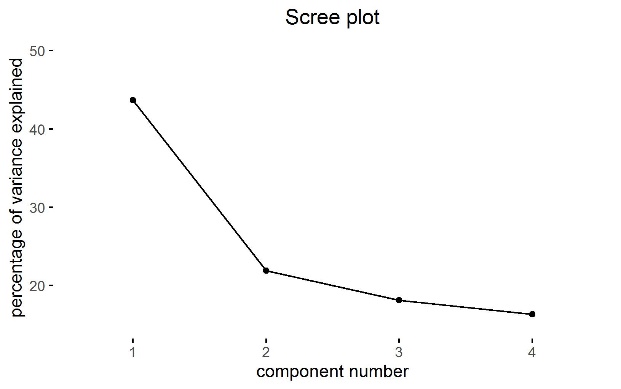

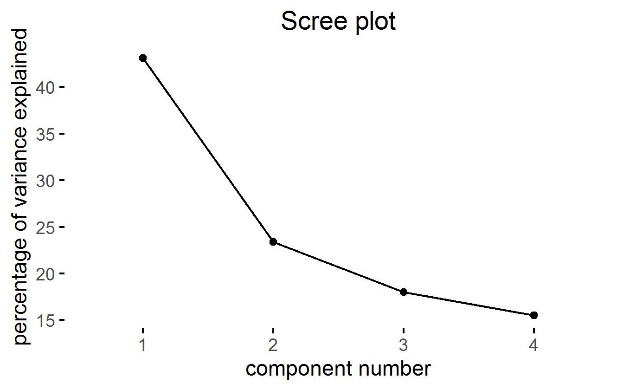
**

**Figure S1**

*Scree plot of PCA on cognition variables in connectome sample*

**Figure S2**

*Scree plot of PCA on cognition variables in dMRI sample*

*PCA white matter*  After applying the consistency thresholding method to retain 30% of all connections, 1071 connections remained. We entered the FA and MD values for all these connections into two separate PCA’s. To calculate the IDP derived gFA and gMD measures, we again entered the FA and MD values of all 27 tracts in two separate PCA’s. The individual loadings on the first unrotated component were then computed and used as a measures of gFA or gMD for both connectome tracts and raw UKB data.


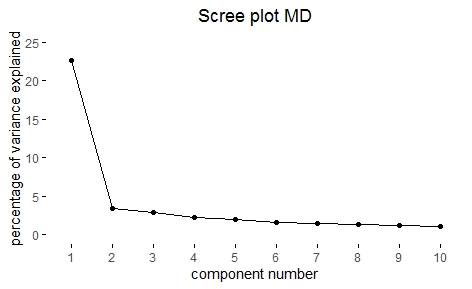
**
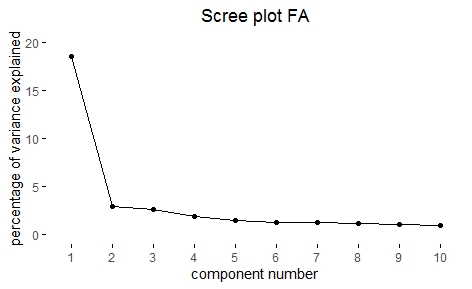
**

**Figure S4**

*Scree plot of PCA on connectome tracts FA*

**Figure S3**

*Scree plot of PCA on connectome tracts MD*

**
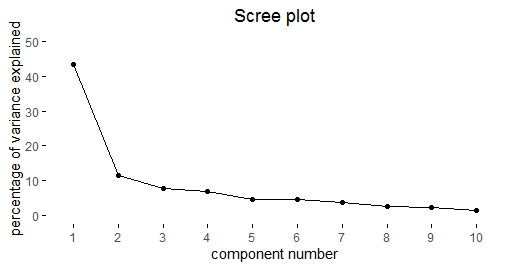
**

**Figure S6**

*Scree plot of PCA on IDP tracts MD, connectome sample*

**Figure S5**

*Scree plot of PCA on IDP tracts FA, connectome sample*

**
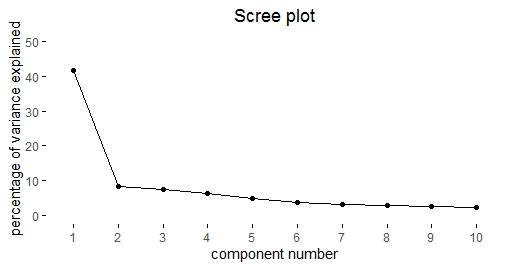
**


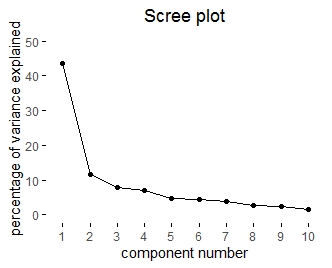
**
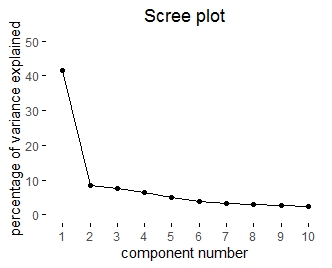
**

**Figure S8**

*Scree plot of PCA on IDP tracts FA, dMRI sample*


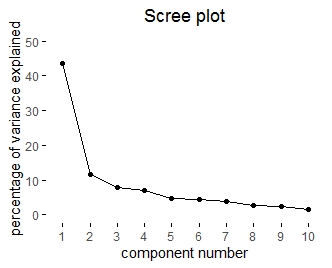


**Figure S7**

*Scree plot of PCA on IDP tracts FA, dMRI sample*


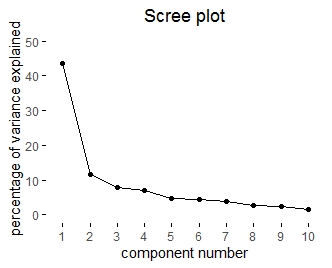

**Table S4**

*Loadings of each IDP tract on the first principal component, per sample*

**Figure S9** The defined SEM model

'**# Direct effect**

AnyPLE ~ c * GeFA + sex + age

g_factor_6170 ~ a * GeFA + sex + age

#**Mediators**

AnyPLE ~ b * g_factor_6170

logRT ~ d * GeFA + sex + age

g_factor_6170 ~ e * logRT

AnyPLE ~ f * logRT

#**Indirect effect**

ab := a*b

de := d*e

df := d*f

sumIDE := (a*b)+(d*f) + (d*e*b)

#**Total effect**

t1 := c +(a*b)

t2 := a +(d*e)

total := c+(a*b) +(d*f) + (d*e*b)'

**Exploratory analyses**

To do the exploratory analyses, we first performed the PCAs for cognition and white matter, as described above and in the article, on the full available sample of N = 25945. We then removed 50 participants without reaction time data, 20 outliers in the PLE group and 240 outliers in the no PLE group. Then we selected the participants in the PLE group with a distress score of 2 or higher. These participants made up the PLE group in the exploratory analyses. All statistical analyses were the same as for the confirmatory analyses.

As suggested by a reviewer, we also conducted the path analysis with 3 outcome groups: no PLE, PLE without distress and PLE with distress. Since this analysis proved to be non-significant for the relation between white matter and PLEs. We than ran several post-hoc analysis, each time selecting two groups, to find out why this result was non-significant. Here, we found that there was a significant relation between white matter and having PLEs without distress versus having PLEs with distress. This supports our original exploratory result that distress is an important factor in these analyses. Supporting tables can be found in tables S25 – S30.

**Supplementary Results**

**Supplementary Tables Connectome sample**

***Table S5*** Results SEM with gFA in connectome sample.

***Table S6*** Results SEM with gMD in connectome sample

***Table S7*** Results SEM with GeFA in connectome sample

***Table S8*** Results SEM with GeMD in connectome sample

***Table S9*** Results SEM with IDP derived gFA in connectome sample

***Table S10*** Results SEM with IDP derived gMD in connectome sample

**Supplementary Tables Connectome sample, participants with schizophrenia included**

***Table S11*** Results SEM with gFA in connectome sample + scz

***Table S12*** Results SEM with gMD in connectome sample + scz

***Table S13*** Results SEM with GeFA in connectome sample + scz

***Table S14*** Results SEM with GeMD in connectome sample + scz

***Table S15*** Results SEM with IDP derived gFA, in connectome sample +scz

***Table S16*** Results SEM with IDP derived gMD, in connectome sample +scz

**Supplementary Tables dMRI sample**

***Table S17*** Results SEM gMD in dMRI sample

***Table S18*** Result SEM gFA in dMRI sample

***Table S19*** Results SEM gMD in dMRI sample + scz

***Table S20*** Results SEM gFA in dMRI + scz

**Supplementary tables, exploratory distress analyses**

***Table S21*** Results exploratory SEM analyses gMD

***Table S22*** Result exploratory SEM analysis gFA

***Table 23*** Results exploratory SEM analyses gMD + scz

***Table 24*** Result exploratory SEM analysis gFA + scz

**Supplementary tables, exploratory analyses with three ogroups**

***Table 25*** Result exploratory SEM analysis gFA ~ no PLE, PLE + distress, PLE – distress

***Table 26*** Result exploratory SEM analysis gMD ~ no PLE, PLE + distress, PLE – distress

**Supplementary tables exploratory analyses PLE + distress vs PLE – distress**

***Table 27*** Result exploratory analysis gFA ~ PLE + distress vs PLE – distress

***Table 28*** Result exploratory analysis gMD ~ PLE + distress vs PLE – distress

**Supplementary tables exploratory analyses no PLE vs PLE – distress**

***Table 29*** Result exploratory analysis gFA ~ no PLE vs PLE – distress

***Table 30*** Result exploratory analysis gMD ~ no PLE vs PLE – distress

**Supplementary References**

Alfaro-Almagro, F., Jenkinson, M., Bangerter, N. K., Andersson, J. L. R., Griffanti, L., Douaud, G., … Smith, S. M. (2018). Image processing and Quality Control for the first 10,000 brain imaging datasets from UK Biobank. *NeuroImage*, *166*, 400–424. https://doi.org/10.1016/j.neuroimage.2017.10.034

Fawns-Ritchie, C., & Deary, I. J. (2019). Reliability and validity of the UK Biobank cognitive tests. *MedRxiv*, 19002204. https://doi.org/10.1101/19002204

Miller, K. L., Alfaro-Almagro, F., Bangerter, N. K., Thomas, D. L., Yacoub, E., Xu, J., … Smith, S. M. (2016). Multimodal population brain imaging in the UK Biobank prospective epidemiological study. *Nature Neuroscience*, *19*(11), 1523–1536. https://doi.org/10.1038/nn.4393
